# Evaluation of the contribution of individual arteries to the cerebral blood supply in patients with Moyamoya angiopathy: comparison of vessel-encoded arterial spin labeling and digital subtraction angiography

**DOI:** 10.1007/s00234-024-03338-7

**Published:** 2024-03-16

**Authors:** Leonie Zerweck, Rolf Pohmann, Uwe Klose, Petros Martirosian, Patrick Haas, Ulrike Ernemann, Nadia Khan, Constantin Roder, Till-Karsten Hauser, Florian Hennersdorf

**Affiliations:** 1 Department of Diagnostic and Interventional Neuroradiology, University Hospital Tuebingen, Hoppe-Seyler-Straße 3, 72076 Tuebingen, Germany; 2 Magnetic Resonance Center, Max-Planck-Institute for Biological Cybernetics, Tuebingen, Germany; 3 Section on Experimental Radiology, Department of Diagnostic and Interventional Radiology, University Hospital Tuebingen, Tuebingen, Germany; 4 Department of Neurosurgery, University Hospital Tuebingen, Tuebingen, Germany; 5 Moyamoya Center, University Children’s Hospital, Zurich, Switzerland

**Keywords:** Moyamoya angiopathy, Vessel-encoded arterial spin labeling, Digital subtraction angiopathy, Cerebral perfusion

## Abstract

**Purpose:**

Vessel-encoded arterial spin labeling (VE-ASL) is able to provide noninvasive information about the contribution of individual arteries to the cerebral perfusion. The aim of this study was to compare VE-ASL to the diagnostic standard digital subtraction angiography (DSA) with respect to its ability to visualize vascular territories.

**Methods:**

In total, 20 VE-ASL and DSA data sets of 17 patients with Moyamoya angiopathy with and without revascularization surgery were retrospectively analyzed. Two neuroradiologists independently assessed the agreement between VE-ASL and DSA using a 4-point Likert scale (no- very high agreement). Additionally, grading of the vascular supply of subterritories (A1-A2, M1-M6) on the VE-ASL images and angiograms was performed. The intermodal agreement was calculated for all subterritories in total and for the subdivision into without and after revascularization (direct or indirect bypass).

**Results:**

There was a very high agreement between the VE-ASL and the DSA data sets (median = 1, modus = 1) with a substantial inter-rater agreement (*k*_*w*_ = 0.762 (95% CI 0.561–0.963)). The inter-modality agreement between VE-ASL and DSA in vascular subterritories was almost perfect for all subterritories (*k* = 0.899 (0.865–0.945)), in the subgroup of direct revascularized subterritories (*k* = 0.827 (0.738–0.915)), in the subgroup of indirect revascularized subterritories (*k* = 0.843 (0.683–1.003)), and in the subgroup of never revascularized subterritories (*k* = 0.958 (0.899–1.017)).

**Conclusion:**

Vessel-encoded ASL seems to be a promising non-invasive method to depict the contributions of individual arteries to the cerebral perfusion before and after revascularization surgery.

## Introduction

Moyamoya angiopathy (MMA) is a cerebrovascular disease characterized by stenosis of the terminal parts of the internal carotid artery (ICA), as well as the proximal branches of the middle cerebral artery (MCA) and the anterior cerebral artery (ACA) [1, 2]. Patients with MMA commonly present with cerebrovascular events such as transient ischemic attacks (TIAs) and ischemic or hemorrhagic strokes [1]. A curative therapy does not exist [1]. A commonly used therapy to prevent ischemic strokes is neurosurgical revascularization. This may be a direct bypass, i.e., direct anastomosis between branch of a donor scalp artery, usually the superficial temporal artery (STA) and a distal branch of a cortical artery (STA-MCA, STA-ACA bypass) or an indirect revascularization where donor artery (EDAS: encephalo-arterio-synangiosis), temporalis muscle, (EMS: encephalo-myo-synangiosis) dura (EDS: encephalo-duro-synangiosis), or galea-periost (EGPS: encephalo-galeaperiost-synangiosis) may be used as a synangiosis triggering neovascularization [1, 3].

After bypass surgery, it is important to assess the collateralization and the contribution of the bypass to the cerebral perfusion [4]. Digital subtraction angiography (DSA) is considered the diagnostic gold standard for preoperative diagnostic confirmation of the disease [1, 5] as well as in examining the postoperative patency of the bypass and its distal territorial filling [1, 4].

In recent years, MRI-based techniques gained importance in assessment of the cerebral perfusion without the need for application of contrast agent [1, 5–9]. Namely, arterial spin labeling (ASL) became increasingly important in the last few years [10–13]. ASL uses blood water as an endogenous tracer by spatially selectively inverting the longitudinal magnetization of the blood supplying the brain [5, 10, 14]. The “labeled” blood leads in the cerebral tissue to a reduced magnetization resulting in a lower signal intensity downstream of the labeling location [5, 10]. By subtracting images read out “after labeling” from control images without inversion of the inflowing blood, the amount of “labeled” blood reaching the brain tissue can be calculated [5, 10].

Vessel-encoded arterial spin labeling (VE-ASL) [15] supplements information on arterial territories by encoding blood from two (or more) selected arteries, maintaining the full sensitivity of pseudo-continuous ASL (PCASL). VE-ASL thus makes it possible to visualize brain regions supplied by selected arteries [16].

The aim of this study was to evaluate the ability of VE-ASL to visualize the contribution of specific arteries to the cerebral blood supply in comparison to the diagnostic gold standard DSA. For this purpose, we compared the selective blood flow of Moyamoya patients without prior bypass surgery and of patients after direct and indirect revascularization.

## Methods

### Patient selection

A retrospective analysis of VE-ASL and DSA data sets of patients with MMA was performed. All imaging scans were performed as routine clinical scans during 2018 and 2023. Inclusion criteria of the study were angiographically proven MMA and the availability of the VE-ASL data and the DSA data at the most 365 days apart with no revascularization therapy in-between. Both preoperative and postoperative patients were included in the study. Exclusion criteria were secondary cerebral diseases or hemodynamically relevant carotid artery stenoses. For a subgroup analysis, each patient’s hemisphere was classified as either neurologically affected or unaffected. The classification was based on patients’ history derived from the electronical medical records. Neurological symptomatic hemispheres were defined as those with either a history of TIAs or persistent neurological deficits (contralateral sensory, motor or language deficits) attributable to a hemisphere. Ethical approval was obtained from the local Ethics Committee (390/2023BO2).

### MRI data acquisition

All MR images were acquired on a 3 T MR Scanner (Magnetom Skyra, Siemens, Erlangen, Germany) using a standard 20-channel head coil. All included patients underwent the standardized MR imaging protocol including among others a VE-ASL sequence, as described in more detail in the following.

Conventional non vessel-selective ASL usually consists of two image types (“tag” and “control” image) that contain identical static tissue signal components but differ in the signal of the inflowing arterial magnetization [15]. Pseudo-continuous (PCASL) is based on a label, and a control cycle where a train of closely spaced RF pulses in conjunction with a synchronously pulsed gradient field is used to invert the magnetization of the blood flowing through the labeling plane [15].

For the vessel-selective data, the PCASL sequence was extended to four cycles, with the two additional cycles used to encode two vessel positions. In those additional scans, only the blood flowing through selected vessels is inverted, leaving the magnetization of blood flowing through the other arteries unchanged [15]. In conjunction with the regular PCASL labeling scheme, this allows to visualize the perfusion territories from the selected arteries only [15].

**Fig. 1:**
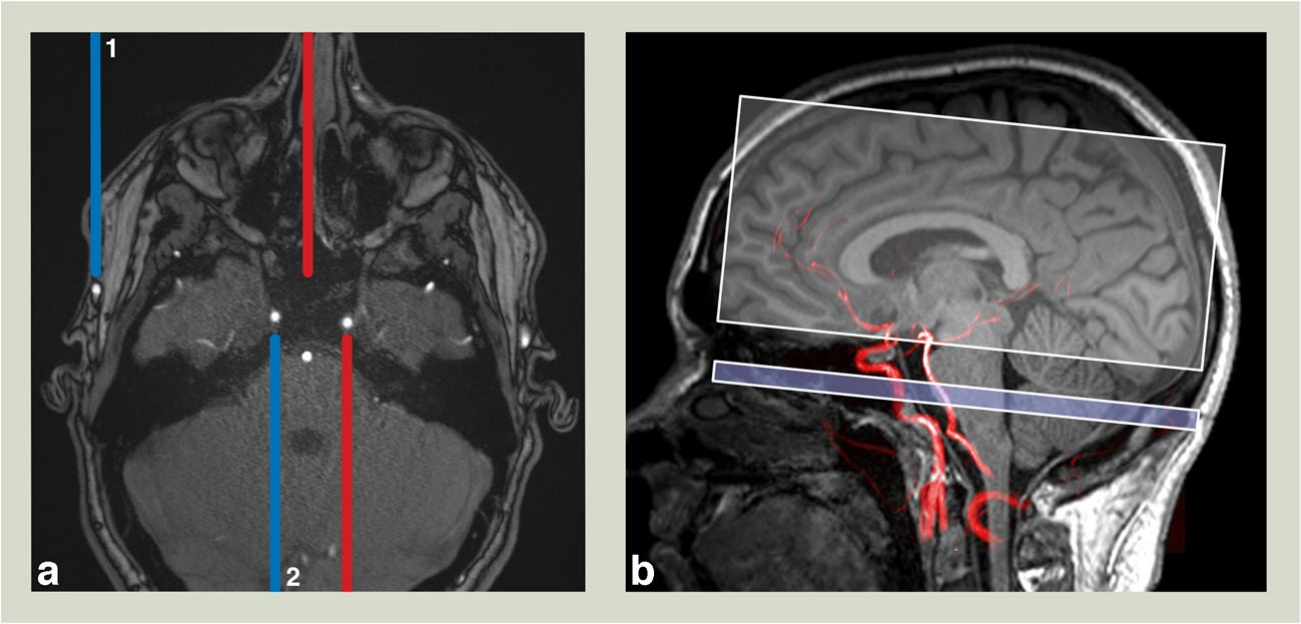
Axial MRI image with an exemplary vessel-encoded arterial spin labeling scheme (**a**). The blue and red lines depict the locations that are placed in contrast. Top (1): tagging of the right superficial temporal artery and a point midway between both internal carotid arteries. Bottom (2): tagging of both internal carotid arteries. Sagittal MRI image with time-of-flight maximum intensity projection (**b**) depicting the labeling position (slim lower bar) and the upstream brain volume (thick upper bar)

For planning of the vessel encoded labeling, a large-coverage time-of -flight (TOF) angiogram was used. In case of prior extracranial-intracranial bypass surgery, the two selected labeling positions were placed at the ipsilateral STA and midway between both ICAs. If no revascularization was performed, both ICAs were labeled. An exemplary VE-ASL labeling scheme depicting the selective tagging can be seen in Fig. 1. The VE-ASL data acquisition was performed with the following parameters: TR = 3700 ms, TE = 21 ms, slices = 12, slice thickness = 6 mm, distance factor = 20%, FoV read = 220 mm, FoV Phase = 100%, labeling duration 1500 ms, and labeling delay = 1500 ms. Sixteen repetitions of the sets of four scans with different label and a M0 scan were acquired within 4:02 minutes. The planning of the vessel labeling was performed by a neuroradiologist with extensive experience in neuroradiological imaging.

### Digital subtraction angiography data acquisition

All DSA data was acquired after selective catheterization and intraarterial injection of iodine contrast agent with a 4-F vessel catheter in the bilateral ICAs, external carotid arteries (ECAs), and the dominant-sided vertebral artery (VA). Anteroposterior and lateral projection angiograms were obtained.

### Data evaluation and statistical analysis

In the first part of the study, the agreement between VE-ASL and DSA with respect to the extent of the territories of the vessel-selectively labeled arteries and the territories of the injected arteries was assessed by two neuroradiologists with extensive experience in neurovascular interventions and imaging (rater 1: 11 years of experience, rater 2 : 21 years of experience) using an equidistant Likert scale ranging from 1 to 4 (1 = very high agreement, 2 = high agreement, 3 = moderate agreement, and 4 = no agreement). Both raters evaluated the data independently and afterwards in consensus.

Modus and median were calculated, as well as inter-rater agreement using quadratic weighted Cohen’s kappa *k*_*w*_.

In the second part of the study, the VE-ASL images and subsequently the angiograms were divided into vascular subterritories (A1-A2, M1-M6), guided by the Alberta Stroke Program Early CT Score (ASPECTS) locations and the arterial territories atlas [17] and evaluated as described in the following:

Following Hwang et al., the VE-ASL images were graded as follows: 0 = no or minimal ASL signal; 1 = moderate ASL signal from the contralateral ICA or the bypass (ECA); 2 = high ASL signal from the contralateral ICA or the bypass (ECA); and 3 = normal (ipsilateral) perfusion [4]. As proposed by Hwang et al. and Zaharchuk et al., the DSA collateral vessel grading was performed as follows: 0 = no collateral vessels visible (absence of any capillary blush); 1 = mild to moderate collateral vessels; 2 = robust collateral vessel flow; and 3 = normal antegrade flow [4, 18]. The rating was performed by rater 1 after a 2-week interval to avoid potential recall bias. The ASL and DSA images were presented in a random order.

The inter-modality agreement between the VE-ASL and the DSA grading was calculated for all patients’ subterritories, as well as for a subgroup of subterritories after bypass-surgery, for a subgroup of subterritories after indirect revascularization and for a subgroup of subterritories without any revascularization surgery using linear weighted Cohen’s *k*.

We performed a subgroup analysis of the inter-modality agreement between the VE-ASL and the DSA grading in subgroups of directly, indirectly, and combined revascularized patients.

Additionally, a further subgroup analysis of the inter-modality agreement between the VE-ASL and the DSA grading in subterritories of neurologically affected hemispheres and neurologically unaffected hemispheres was performed.

Interpretation of the *k* values was as follows: 0–0.20 slight agreement; 0.21–0.40 fair agreement; 0.41–0.60 moderate agreement; 0.61–0.80 substantial agreement and 0.81–1 almost perfect agreement [4, 19].

All statistical analyses were performed using SPSS Statistics (IBM Corp. Released 2021. IBM SPSS Statistics for Windows, Version 28.0. Armonk, NY: IBM Corp). *p* values below 0.05 were considered as significant.

## Results

### Participants

General patient data can be found in Table 1. In total, 20 VE-ASL and DSA data sets of 17 individual patients with MMA fulfilled the inclusion criteria. We included 6 data sets of patients with unilateral revascularization surgery, 8 data sets of patients with bilateral revascularization surgery, and 6 data sets of patients without prior surgery. Of the 14 patients with revascularization surgery, 8 patients were directly revascularized, 6 patients were combined directly, and indirectly revascularized and no patient was only indirectly revascularized. In total, 108 subterritories with direct bypass surgery, 18 subterritories with indirect revascularization surgery, and 122 subterritories without revascularization surgery were included. Each directly revascularized territory included in the study underwent STA-MCA bypass surgery. Indirect revascularization was performed in ACA territories and in on case in a MCA territory due to inadequate recipient vessel.

**Table 1:** General patient data

Individual patients (*n*)	17
Median age (range)	39 (17–51)
Female: male ratio	3.25 : 1
VE-ALS data sets	20
DSA data sets	20
Data sets without revascularization surgery	6
Data sets after direct revascularization surgery	8
Data sets after indirect revascularization surgery	0
Data sets after combined direct and indirect revascularization surgery	6
Data sets after unilateral revascularization surgery	8
Data sets after bilateral revascularization surgery	6
Subterritories without revascularization surgery	122
Subterritories after direct revascularization surgery	108
Subterritories after indirect revascularization surgery	18
Days between MRI and DSA data acquisition (median, range)	1 (0–309)
Days between revascularization and VE-ALS data acquisition (median, range)	329 (27–1085)
Days between revascularization and DSA data acquisition (median, range)	366 (2–1086)
Territories of a neurologically symptomatic hemisphere	152
Territories of a neurologically asymptomatic hemisphere	96

### Assessment of the agreement between VE-ASL and DSA with respect to the extent of vascular territories

The evaluation of both raters revealed a very high agreement between VE-ASL and DSA on a Likert scale with a median of 1 and a modus of 1. The contingency table is seen in Table 2. The inter-rater agreement was substantial (*k*_*w*_ = 0.762, 95% confidence interval (CI) 0.561–0.963, *p* < 0.001). The rating in consensus of both raters resulted in 15/20 (75.0%) data sets in a very high agreement, in 3/20 (15.0%) data sets in a high agreement, in 2/20 (10.0%) in a moderate agreement, and in 0/20 (0%) data sets in no agreement between VE-ASL and DSA (see Table 3).

**Table 2:** Contingency table for the agreement between VE-ASL and DSA with respect to the extent of vascular territories rated by rater 1 and rater 2

		Rater 1				
		Very high agreement	High agreement	Moderate agreement	No agreement	Total
Rater 2	Very high agreement	14	2	0	0	16
	High agreement	2	0	0	0	2
	Moderate agreement	0	0	0	0	0
	No agreement	0	0	2	0	2
	Total	16	2	2	0	20

**Table 3:** Consensus rating of the agreement between VE-ASL and DSA respective the extent of vascular territories

	Frequency (%)
Very high agreement	15 (75%)
High agreement	3 (15%)
Moderate agreement	2 (10%)
No agreement	0 (0%)

### Evaluation of vascular subterritories with respect to the blood supply

Analysis of the inter-modality agreement between VE-ASL and the DSA in vascular subterritories (A1-A2, M1-M6) was almost perfect with a linear weighted *k* of 0.899 (95% CI 0.853–0.945; *p* < 0.001) (see Table 4). The inter-modality agreement between the VE-ASL and the DSA data sets was significant in all individual patients (see Table 4). The patient specific inter-modality agreement ranged from a linear weighted k of 0.535 (95% CI 0.114–0.956; *p* = 0.006) to a linear weighted *k* of 1.000 (95% CI 1.000–1.000; *p* < 0.001) (see Table 4).

**Table 4:** Agreement between the VE-ASL and the DSA rating in vascular subterritories

Patient number	All subterritories: Linear weighted *k* (95% confidence interval) *p*-value	Subgroup of directly revascularized territories: Linear weighted *k* (95% confidence interval) *p*-value	Subgroup of indirectly revascularized territories: Linear weighted *k* (95% confidence interval) *p*-value	Subgroup of not revascularized territories: Linear weighted *k* (95% confidence interval) *p*-value
1a	1.000 (1.000–1.000) < 0.001	1.000 (1.000–0.000) < 0.001	–	n/a
1b	1.000 (1.000–1.000) < 0.001	1.000 (1.000–1.000) < 0.001	–	n/a
2	1.000 (1.000–1.000) < 0.001	–	–	1.000 (1.000–1.000) < 0.001
3	1.000 (1.000–1.000) < 0.001	–	–	1.000 (1.000–1.000) < 0.001
4	1.000 (1.000–1.000) < 0.001	–	–	1.000 (1.000–1.000) < 0.001
5	0.714 (0.500–0.929) < 0.001	0.647 (0.386–0.909) < 0.001	0.716 (0.191–1.237) 0.082	–
6	0.800 (0.569–1.031) < 0.001	0.500 (0.017–0.983) 0.079	–	1.000 (1.000–1.000) 0.046
7	1.000 (1.000–1.000) 0.005	1.000 (1.000–1.000) 0.014	–	1.000 (1.000–1.000) 0.157
8a	0.846 (0.558–1.135) < 0.001	–	*–*	0.846 (0.558–1.135) < 0.001
8b	1.000 (1.000–1.000) 0.005	1.000 (1.000–1.000) 0.014	n/a	–
9	1.000 (1.000–1.000) < 0.001	–	–	1.000 (1.000–1.000) < 0.001
10a	0.756 (0.331–1.199) 0.002	0.571 (−0.121–1.264) 0.121	−0.333 (−0.949 to −0.283) 0.157	1.000 (1.000–1.000) 0.157
10b	0.756 (0.331–1.199) 0.002	0.759 (0.316–1.201) 0.004	1.000 (1.000–1.000) 0.157	–
11	0.686 (0.376–0.997) < 0.001	0.400 (−0.297–1.079) 0.174	0.400 (−0.297–1.079) 0.174	1.000 (1.000–1.000) 0.006
12	1.000 (1.000–1.000) < 0.001	1.000 (1.000–1.000) < 0.001	–	1.000 (1.000–1.000) 0.015
13	0.535 (0.114–0.956) 0.006	0.571 (0.130–1.013) 0.015	–	n/a
14	1.000 (1.000–1.000) < 0.001	–	–	1.000 (1.000–1.000) < 0.001
15	0.771 (0.478–1.065) 0.003	0.714 (0.327–1.102) 0.014	–	n/a
16	1.000 (1.000–1.000) 0.005	1.000 (1.000–1.000) 0.014	–	n/a
17	1.000 (1.000–1.000) 0.005	1.000 (1.000–1.000) 0.014	1.000 (1.000–1.000) 0.157	
Total	0.899 (0.853–0.945) < 0.001	0.827 (0.738–0.915) < 0.001	0.843 (0.683–1.003) < 0.001	0.958 (0.899–1.017) < 0.001

**Fig. 2:**
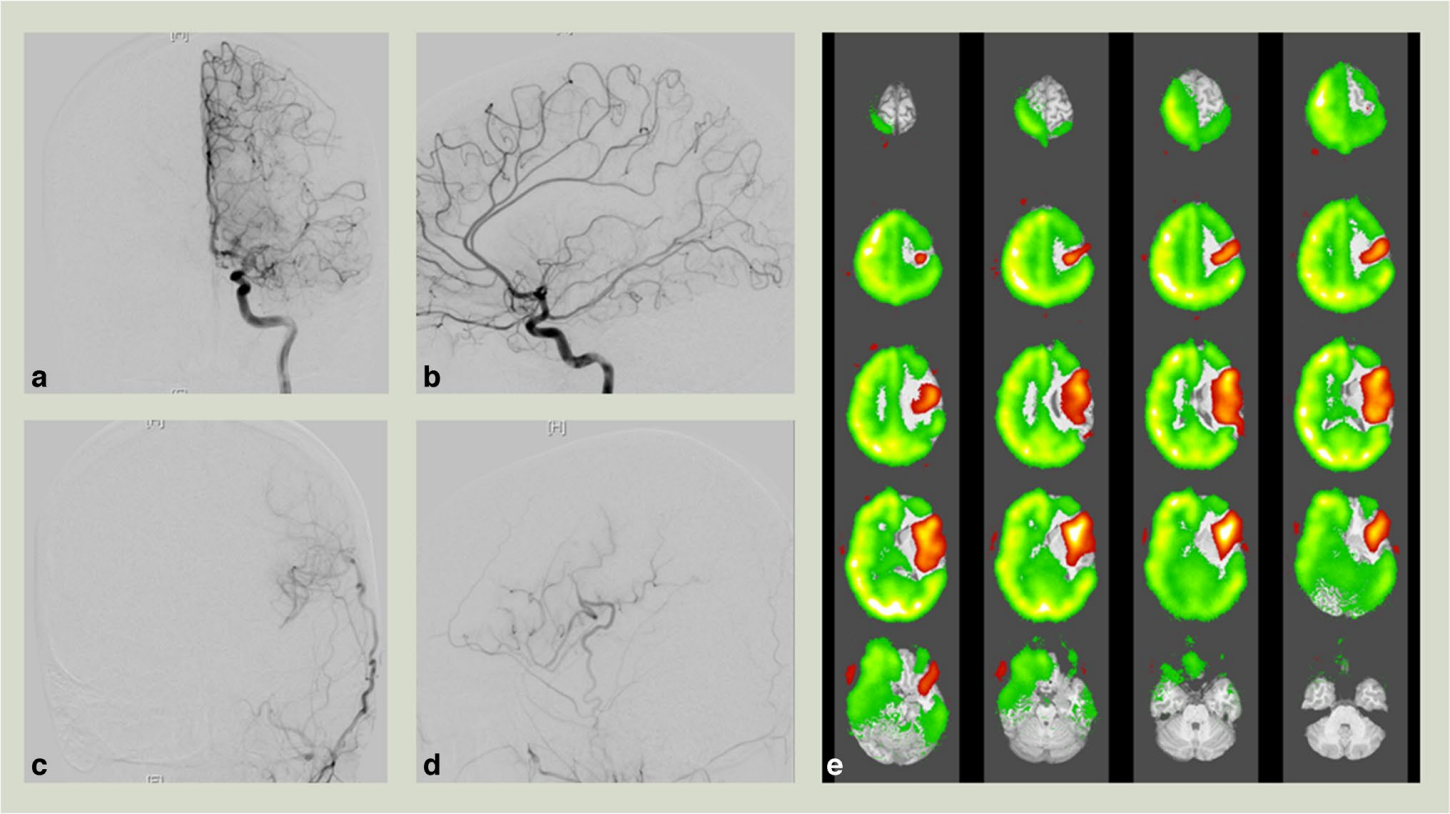
Exemplary digital subtractions angiography (DSA) (**a–d**) and vessel-encoded arterial spin labeling (VE-ASL) (**e**) images with high inter-modality agreement (linear weighted *k* = 1.000 (95% confidence interval = 1.000–1.000; *p* < 0.001) of an 18-year old patient with Moyamoya Angiopathy 15 months after left superficial temporal artery–middle cerebral artery (MCA) bypass surgery. Anteroposterior (**a**) and lateral (**b**) projections of the left internal carotid artery (ICA) DSA reveal normal antegrade flow in the subterritories of the anterior cerebral artery (ACA) and the M3, M4, and M6 subterritories of MCA. Anteroposterior (**c**) and lateral (**d**) projections of the left external carotid artery (ECA) show a high-caliber bypass with robust collateral flow in the M1, M2, and M5 subterritories. Color-coded (VE-ASL) (green: right and left ICA, red: left ECA) depicts normal ICA signal in the right ACA and MCA territories and the left M3, M4 and M6 subterritories, while in the left M1, M2, and M5 subterritories high signal from the left ECA is seen

The inter-modality agreement in the subgroup analysis of only directly revascularized subterritories was almost perfect with a linear weighted k of 0.827 (95% CI 0.738–0.915; *p* < 0.001) (see Table 4). The minimum patient specific inter-modality agreement revealed a linear weighted k of 0.571 (95% CI −0.121–1.264; *p* < 0.121) and the linear weighted k of the maximum patient specific inter-modality agreement was 1.000 (95% CI 1.000–1.000; *p* < 0.001) (see Table 4). Exemplary VE-ASL images and angiograms of a patient after bypass surgery can be seen in Fig. 2.

The inter-modality agreement in the subgroup analysis of indirectly revascularized subterritories was almost perfect with a linear weighted *k* of 0.843 (95% CI 0.683–1.003; *p* < 0.001) (see Table 4). The linear weighted k of the minimum patient specific inter-modality agreement was −0.333 (95% CI −0.949 to −0.283; *p* = 0.157) and the linear weighted *k* of the maximum patient specific inter-modality agreement was 1.000 (95% CI 1.000–1.000; *p* = 0.157) (see Table 4). It should be considered that the subgroup of indirectly revascularized subterritories was very small (*n* = 22) and that in some patients only the ACA territory of one hemisphere was indirectly revascularized and therefore only 2 subterritories were included in the patient specific analysis.

The inter-modality agreement in the subgroup analysis of never vascularized subterritories was almost perfect and revealed a linear weighted *k* of 0.958 (95% CI 0.899–1.1007; *p* < 0.001) (see Table 4). The patient specific inter-modality agreement ranged from a linear weighted *k* of 0.846 (95% CI 0.558–1.135; *p* < 0.001) to a linear weighted *k* of 1.000 (95% CI 1.000–1.000; *p* < 0.001) (see Table 4).

The inter-modality agreement in the subgroup of directly revascularized patients was almost perfect (linear weighted *k* = 0.868 (95% CI 0.778–0.959; *p* < 0.001)), as well as in the subgroup of combined revascularized patients (linear weighted *k* = 0.822 (95% CI 0.723–0.902; *p* < 0.001)) and the subgroup of never revascularized patients (linear weighted *k* = 0.966 (95% CI 0.901–1.032; *p* < 0.001)). A subgroup analysis of indirectly revascularized patients could not be performed, because no patient was only indirectly revascularized.

The inter-modality agreement in the subgroup analysis of subterritories of neurologically symptomatic hemispheres and subterritories of neurologically asymptomatic hemispheres was almost perfect in both cases with a linear weighted *k* of 0.909 (0.859–0.960; *p* < 0.001) and of 0.856 (0.745–0.967; *p* < 0.001).

## Discussion

Vessel selective ASL was proposed as a noninvasive MRI based method to depict the vascular territory of a single selected artery [4]. The technique used here has the advantage of being able to simultaneously separate two arterial perfusion territories without loss in sensitivity. Past studies revealed a good intermodally agreement between other selective ASL techniques and computed tomography angiopathy (CTA) [20] and TOF angiography [21] in patients with MMA.

The aim of this study was to validate VE-ASL in patients with MMA regarding the capability to depict the contribution of specific vessels to the cerebral blood supply in comparison to the diagnostic gold standard DSA.

In this study, two raters independently and consensually rated the extent of vascular territories on a 4-point Likert scale very similarly with a substantial inter-rater agreement. We performed an additional analysis of vascular subterritories with respect to the perfusion status and differentiated between absent perfusion, mild to moderate perfusion and robust perfusion via collateral vessels from a bypass or the supplying artery of the contralateral hemisphere, and normal ipsilateral perfusion. The inter-modality agreement between VE-ASL and DSA in the subterritory analysis was almost perfect for all subterritories, in the subgroup of direct revascularized subterritories, in the subgroup of indirect revascularized subterritories and in the subgroup of never revascularized subterritories. In all subgroups of directly, combined and never revascularized patients the inter-modality agreement was almost perfect. The almost perfect agreement was shown, regardless of whether a history of TIAs or persisting neurological symptoms were present or not.

The study suggests that VE-ASL might be a good method to show the contribution of a specific vessel to the cerebral perfusion and is in accordance with previous studies exploring the potential of VE-ASL [4, 16, 20, 22–24].

As the signal-to-noise ratio is the limiting factor in most ASL applications, using a PCASL-based technique with its superior sensitivity is an important factor in the study, especially, as it can be relatively easily enhanced to include labeling of different vessels. One further advantage of the VE-ASL technique we used in this study is the relative short acquisition time (up to four vessels in 4:02 min).

The estimation of the extent of individual vascular territories is highly relevant in patients with MMA, especially after revascularization surgery to depict the contribution of a bypass [4, 5]. Therefore, VE-ASL seems to be a promising tool to monitor revascularization areas in follow up examinations [4, 5].

In addition to MMA, there are other fields of application in which mapping of vascular territories by use of VE-ASL may be relevant. For example in the etiological classification of strokes, VE-ALS might be helpful to identify whether multiple ischemic lesions are located in the same vascular territory and to distinguish between cardioembolic events and a vascular stenosis as etiology of stroke [11, 25, 26]. In ischemic strokes, it can be difficult to distinguish the brain-feeding arteries of the affected vascular territory [25]. This is due to the wide range of common variabilities and anomalies in the vascular territories of cerebral arteries and particularly relevant in patients with cerebrovascular diseases [25, 26]. According to Hendrikse et al. additional information from the territorial ASL images can change infarct classifications in 11% of cortical or border zone infarcts supporting the value of ASL in clinical practice [25]. Accurate prediction of vascular territories could also have clinical value for neurosurgical planning, e.g., for aneurysm clipping or tumor resection [11, 27].

One big advantage of VE-ASL is the non-invasiveness and the lack of radiation exposure or the use of contrast agents [5, 11]. For this reason, it offers the advantage that it can be repeated as often as desired and is therefore particularly suitable for children and patients allergic to contrast agent or with renal insufficiency [5, 11, 21, 22]. Another advantage of VE-ASL is that it provides three-dimensional cross-sectional images, allowing accurate localization of vascular territories more easily than with the overlapping projection images on angiograms [4, 22].

Nevertheless, there are also disadvantages of VE-ASL. One limitation of VE-ASL is the difficulty of cerebral blood flow quantification [4, 20]. Another disadvantage of VE-ASL over DSA is that in case of direct and indirect revascularization surgery, it is not possible to distinguish between perfusion via the direct and indirect bypass components [4] and the patency of anastomoses and the single collateral vessels are not directly visualized.

VE-ASL as performed in this study does not provide information about the cerebral perfusion reserve/cerebral reactivity, which is considered as a surrogate parameter for the risk of stroke and plays an important role in the indication of revascularization in patients with MMA [6, 9, 28, 29]. Previous studies showed that it is principally possible to estimate the cerebral perfusion reserve/cerebral reactivity with ASL by administrating vasoactive agents (such as acetazolamide or inhaled CO_2_) [11, 13, 30–32]. An open question so far is whether this is vessel-selectively possible by use of VE-ASL.

In addition to the intrinsic limitations of the VE-ASL technique mentioned above, our study had limitations. Due to the low prevalence of MMA one limitation was the limited sample size, which involved the risk of low statistical power and which allowed in the subgroup analysis of indirectly revascularized territories only tentative results. Another limitation was that only territories of the ACA and the MCA were evaluated. Although the arteries of the posterior circulation are rarely affected in MMA, neo-vascularization and collateral pathways may also have an impact on the posterior circulation and should be considered in future studies. This proof-of-principle study showed that VE-ASL seems to be a promising noninvasive method to distinguish 3-dimensionally the origin of feeding arteries of a vascular territories. Future prospective studies should validate VE-ASL immediately before and repeatedly after surgery and compare it with the diagnostic standard DSA.

## Conclusion

Vessel-encoded ASL agrees well with the diagnostic standard DSA in depicting the contribution of selective arteries of patients with MMA. It seems to be a promising non-invasive method to visualize the contribution of individual arteries to the cerebral perfusion before and after revascularization surgery.

## Data Availability

The data that support the findings of this study are available from the corresponding author, upon reasonable request.
